# From amenorrhea to pregnancy: spontaneous recovery of the female athlete triad during the COVID-19 pandemic

**DOI:** 10.20945/2359-4292-2026-0007

**Published:** 2026-01-28

**Authors:** Eduardo Medeiros Ferreira da Gama, Francisco de Paula Paranhos-Neto, Miguel Madeira, Laura Maria Carvalho Mendonça, Maria Lucia Fleiuss Farias

**Affiliations:** 1 Divisão de Endocrinologia, Hospital Universitário Clementino Fraga Filho, Universidade Federal do Rio de Janeiro, Rio de Janeiro, RJ, Brasil; 2 Divisão de Reumatologia, Hospital Universitário Clementino Fraga Filho, Universidade Federal do Rio de Janeiro, Rio de Janeiro, RJ, Brasil

**Keywords:** Female athlete triad syndrome, relative energy deficiency in sport, amenorrhea, bone density

## Abstract

The Female Athlete Triad (Triad) and Relative Energy Deficiency in Sport (RED-S) are conditions associated with low energy availability (EA) that can lead to menstrual dysfunction, impaired bone health, and metabolic disturbances. This case report describes the remission of Triad in a professional triathlete during the COVID-19 pandemic. A 24-year-old female triathlete was evaluated in 2018. She reported bulimia nervosa from 12–15 years old and hypothalamic amenorrhea since she was 21 years old. Her training volume was approximately 20 hours per week (swimming, cycling, and running), and the amount of EA was critically low (< 10 kcal/fat free mass/day). The athlete had lower than expected BMD at DXA scan (lumbar spine Z-score -2.3 SD) and lower cortical and trabecular vBMD and trabecular number by comparison with HR-pQCT normative data for young Brazilian women. During the 2020 pandemic, training centers were closed, leading to a decrease in exercise volume and a more balanced energy intake. Menstrual cycles resumed and she conceived spontaneously in the same year. Pregnancy and postpartum recovery were uneventful. She returned to competitions post pandemic, but no longer as a professional athlete. Chronic energy deficiency significantly affects the hypothalamic-pituitary-gonadal axis and bone health. A forced reduction in training intensity and improved dietary intake were key factors in the restoration of menstrual function and reproductive health. This case highlights the potential reversibility of the Female Athlete Triad when energy balance is restored. Awareness and early intervention are essential for preventing long-term consequences in female athletes.

## INTRODUCTION

The Female Athlete Triad (Triad) was initially described as being associated with eating disorders, amenorrhea, and osteoporosis. Over time, this concept has evolved into the broader model of Relative Energy Deficiency in Sport (RED-S), which considers a wide range of systemic effects, such as endocrine, metabolic, and immune dysfunctions. Energy availability (EA) can be estimated as dietary intake minus exercise energy expenditure normalized to lean body mass or fat free mass (FFM). Low EA is a primary driver of these conditions, leading to repercussions on reproductive function and bone health ^(1,2)^.

In high-performance sports, female athletes often prioritize an optimal body composition for performance, which can result in chronic energy deficits. Less than 30 kcal/FFM/day may be insufficient to maintain menstrual function, whereas adequate EA is estimated as > 45 kcal/FFM/day ^(3,4)^.

This case report describes a professional triathlete with the Female Athlete Triad for several years, which was characterized by amenorrhea and changes in bone density and microstructure. This athlete then developed spontaneous remission during the COVID-19 pandemic, which resulted in the return of menstrual function and a subsequent pregnancy.

## CASE PRESENTATION

This patient was included in 2018 in a broad research project of Female Athletes approved by the Ethics Committee of HUCFF-UFRJ (CAAE: 64516117.9.0000.5257). Written informed consent was obtained from all volunteers who participated in the study.

The athletic career began when she was 16 years old, transitioning to professional status by age 20. The training regimen included 20 hours per week distributed among swimming (20 km), cycling (10–12 hours), and running (60 km). Despite achieving excellent competition results, she expressed persistent dissatisfaction with her body weight and felt that it did not align with elite competition standards. At age 21, she developed oligomenorrhea and subsequent hypothalamic amenorrhea. She had a prior history of bulimia nervosa (ages 12–15) but denied the use of hormonal contraceptives, tobacco products, alcohol, or illicit drugs.

She was 24 years old during the study, reported amenorrhea since 22 years old and denied previous fractures. A full clinical, anthropometric, metabolic and imaging assessment was performed.

**Figure 1:**
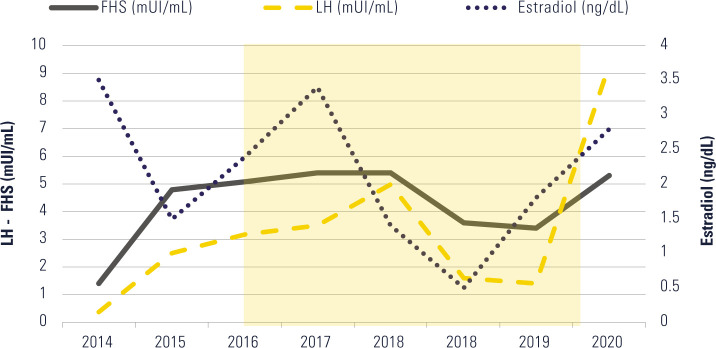
Longitudinal evolution of gonadotropic and ovarian hormones (LH, FSH, and estradiol) from 2014 to 2020. The progressive increase in estradiol concentrations paralleled the normalization of gonadotropins (FSH and LH) following the restoration of energy availability. The shaded area represents the amenorrheic period. Menstrual recovery occurred after a reduction in training volume and improvement in caloric intake.

**Biochemistry:** Gonadotropins, estradiol, and prolactin levels were measured on multiple occasions during the period of menstrual dysfunction, and results consistently demonstrated normal prolactin levels. This endocrine profile is characteristic of functional hypothalamic amenorrhea secondary to low energy availability, as illustrated in Figure 1. The patient's fasting glucose concentration was 96 mg/dL (70-99), vitamin B_12_ concentration was 786 pg/mL (210-980), and folic acid concentration was 18.4 ng/mL (≥ 3.9). Other biochemical parameters, such as follow-up measurements obtained after menstrual recovery, are summarized in Table 1.

**Table 1:** Laboratory data obtained during the study visits conducted while the patient was under functional hypothalamic amenorrhea ("Amenorrheic period") and after menstrual and hormonal recovery in 2020 ("Recovery"). Reference ranges correspond to standard values for women of reproductive age

Parameter (unit)	Amenorrhea (2018)	Recovery (2020)	Reference range
FSH (mIU/mL)	3.6	5.3	3.5–12.5
LH (mIU/mL)	1.6	9.3	2.4–12.6
Estradiol (ng/mL)	<0.5	2.8	30–120 Follicular
Prolactin (ng/mL)	7.0	13.9	5-25
Testosterone (ng/dL)	23	24	8.4–48.1
Insulin (µU/mL)	2.0	5.3	2–25
HOMA-IR	0.4	1.1	< 2.5
IGF-1 (ng/mL)	160	183	110–270
TSH (µIU/mL)	1.8	2.61	0.4–4.0
Free T_4_ (ng/dL)	1.2	1.14	0.8–1.8
Reverse T_3_ (ng/dL)	16	18.2	10–24
Vitamin D (25OHD, ng/mL)	32	40.0	≥20
PTH pg/dL	29	29	15–65
Calcium (mg/dL)	9.4	9.0	8.6–10.0
Phosphorus (mg/dL)	3.6	3.5	2.5–4.5
Magnesium (mg/dL)	1.8	2.0	1.6–2.6

**Dual energy absorptiometry (DXA)** was used to evaluate areal bone mineral density (BMD, g/cm^2)^ and Z-scores (differences between the patient's BMD and age-gender-matched healthy individuals) at the lumbar spine and hip, using a GE Lunar Prodigy Advance densitometer. In accordance with the 2023 International Society for Clinical Densitometry (ISCD) guidelines, a Z-score at or below -2.0 SD indicates "lower than expected BMD". Whole-body DXA scans were used to assess body composition, including total and regional fat mass and fat-free mass (FFM), following standard ISCD procedures ^(5)^. Results were as follows: body mass index = 20 kg/m^2^ (normal range 18.5-24.9), total fat mass = 11.42 kg, fat mass index (total fat/height^2)^ = 3.93 (NR 5-9) and fat free mass = 46.83 kg. Additionally, lumbar spine BMD = 0.893 g/cm^2^ (LS Z-score -2.3 SD), and total hip BMD = 0.953 g/cm^2^ (TH Z-score -0.3 SD).

**High resolution peripheral quantitative computed tomography (HR-pQCT)** was performed using a Scanco Xtreme CT, with a voxel size of 82 µm, and a standard distal radius and tibia protocol. Volumetric BMD and bone microstructure were measured at cortical and trabecular compartments, according to a recent consensus ^(6)^. All data were compared with normative data defined for age-matched Brazilian women, as published by Alvarenga and cols. ^(7)^. This athlete had lower than expected values for volumetric bone mineral density (vBMD) in the tibia, cortical and trabecular vBMD in the radius, and cortical vBMD in the tibia, as well as a lower trabecular number and greater trabecular separation in the radius (Table 2).

**Table 2:** Bone assessment using HR-pQCT at baseline

Variable	Athlete (24.8 yrs)	Reference – Alvarenga (20–29 yrs)
**Distal Radius**		
Total vBMD (mg HA/cm^3)^	352.9	331 (279–370)
Cortical vBMD (mg HA/cm^3)^	**955.8**	1,017 (974–1,053)
Cortical Thickness (mm)	0.93	0.86 (0.72–1.03)
Trabecular vBMD (mg HA/cm^3)^	**136.9**	172 (155–193)
Trabecular Number (1/mm)	**1.49**	2.02 (1.92–2.17)
Trabecular Thickness (mm)	0.077	0.069 (0.064–0.075)
Trabecular Separation (mm)	**0.595**	0.423 (0.387–0.454)
**Distal Tibia**		
Total vBMD (mg HA/cm^3)^	**304.0**	324 (309–365)
Cortical vBMD (mg HA/cm^3)^	**937.3**	1,015 (989–1,043)
Cortical Thickness (mm)	1.29	1.28 (1.05–1.43)
Trabecular vBMD (mg HA/cm^3)^	159.6	168 (152–194)
Trabecular Number (1/mm)	1.72	1.84 (1.61–2.03)
Trabecular Thickness (mm)	0.077	0.077 (0.072–0.088)
Trabecular Separation (mm)	0.503	0.466 (0.416–0.527)

The **Eating Attitudes Test (EAT-26), Bulimic Investigatory Test of Edinburgh (BITE), and Body Shape Questionnaire (BSQ)** were applied as validated Portuguese self-administered instruments to screen for disordered eating and body-image concerns. Results were as follows: EAT-26 = 37 points (positive); BITE = 27 points (positive); BSQ = 118 points (negative).

**Assessment of energy availability:** To estimate the athlete's daily energy consumption, a three-day food record was requested and, include two weekdays and one weekend day. Dietary data were analyzed using computer-based nutrition software (DietWin Personal). The average daily energy intake was then compared with international recommendations to determine whether the intake met the thresholds for adequate EA (≥45 kcal/kg FFM/day) or was critically low (<30 kcal/kg FFM/day) ^(8,9)^. It was determined that the patient's EA was critically low (< 10 kcal/FFM/day).

**Nutritional Intake, Resting Metabolic Rate and Energy Expenditure Evaluation.** The resting metabolic rate and total energy expenditure were estimated by indirect calorimetry to be 1403 kcal/day and 3241 kcal/day, respectively, whereas the patient average daily caloric intake was 2051 kcal/day, which confirmed a significant energy deficit. The distribution of macronutrients consisted of 49% carbohydrates (4.1 g/kg/day), 22% protein (2.4 g/kg/day), and 30% lipids. The daily calcium intake was 673 mg, which is below the recommended intake for optimal bone health.

**Cardiopulmonary exercise testing** (CPET) revealed a maximal oxygen uptake (VO_2_ max) of 58.9 mL/kg/min, which corresponded to 146% of the predicted value. The achieved maximum metabolic equivalent (MET) was 16.8 and, indicated excellent aerobic capacity despite the energetic and hormonal imbalance.

**Figure 2:**
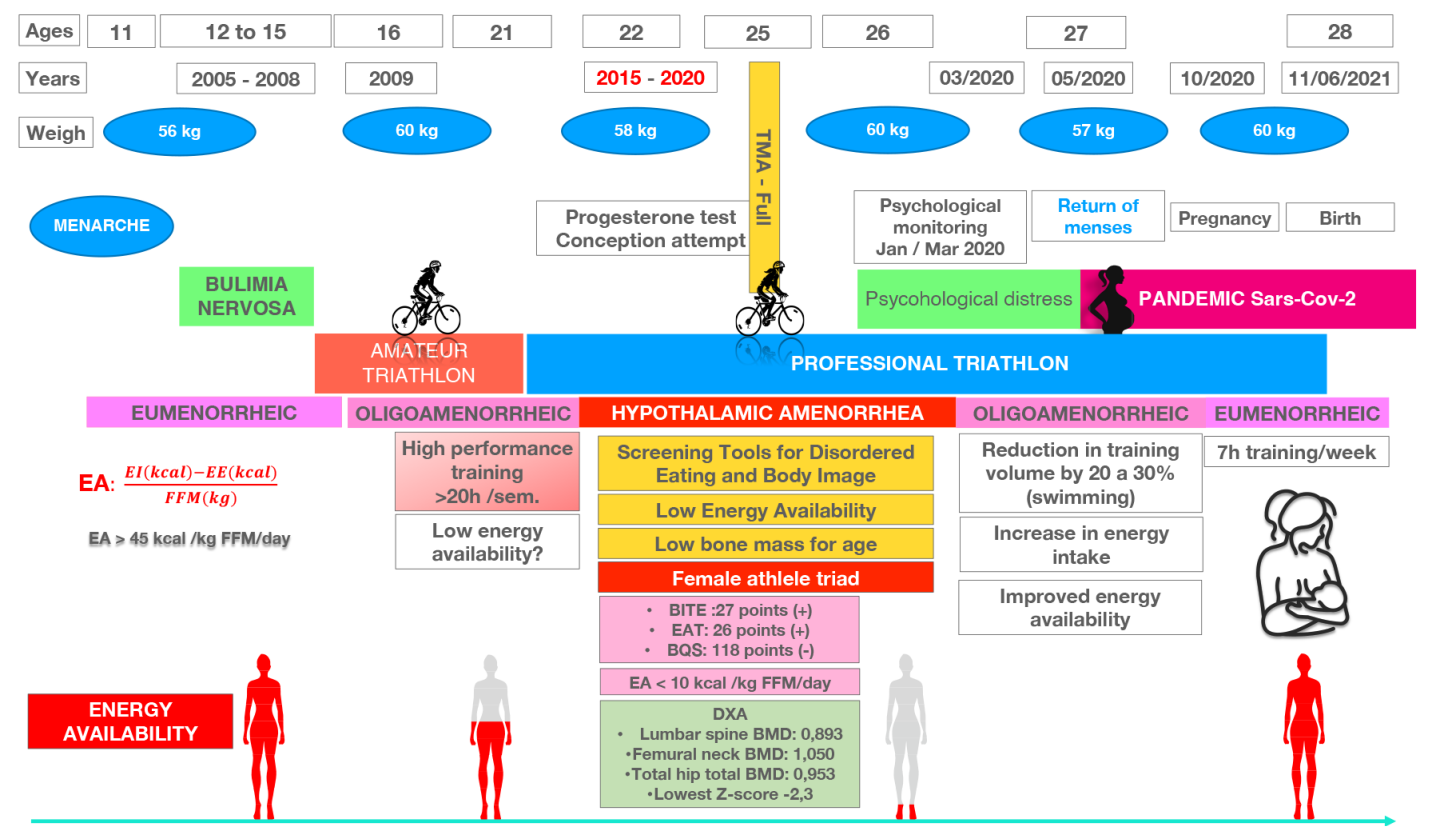
Timeline of the athlete's clinical and training history showing menstrual status, body mass evolution, and major life events. The figure highlights the period of hypothalamic amenorrhea (2015–2020), pandemic-related reduction in training volume, and spontaneous menstrual and reproductive recovery in 2020.

Abbreviations: EA: energy availability; EI: energy intake; EE: energy expenditure; FFM: fat-free mass; BITE: Bulimic Investigatory Test of Edinburgh; EAT: Eating Attitudes Test; BSQ: Body Shape Questionnaire; DXA: dual-energy X-ray absorptiometry.

**Seven-year follow-up:** The patient's body mass showed only minor variations throughout follow-up and, ranged from 56 kg at age 12 to 60 kg at age 26. At the onset of hypothalamic amenorrhea, the patient weighed was 58 kg. During the COVID-19 pandemic, the closure of training centers and cancellation of competitions led to a forced reduction in exercise intensity. The overall training volume markedly decreased – from 15–18 hours to approximately 6–8 hours per week. This, combined with a less restrictive dietary pattern, resulted in an improved energy balance despite no significant weight change. Psychological distress was also noted initially, and required counseling support. In May 2020, after more than five years of amenorrhea, her menstrual cycle resumed, and conception occurred in October, when she weighted 60 kg. This, suggests that the restoration of energy balance, rather than weight gain, was the key determinant of hypothalamic–pituitary–gonadal axis recovery. The pregnancy was uneventful and the baby was born healthy and full-term. Notably, the patient had attempted to conceive four years earlier, during the amenorrheic phase, but there was no withdrawal bleeding after oral progesterone challenge, which confirmed persistent functional hypothalamic suppression. The case is summarized in Figure 2.

She resumed training postpandemic and began participating in competitive triathlon but no longer as a professional athlete. Her menstrual cycles have remained regular since this time, and she had no fractures until 2025.

## DISCUSSION

Chronic low levels of EA have profound effects on the hypothalamic-pituitary-gonadal axis and, lead to menstrual dysfunction and impaired bone health ^(4)^. This case illustrates these changes and shows how spontaneous energy balance restoration through reduced exercise volume and improved dietary intake can reverse these effects.

Laboratory findings throughout the follow-up confirmed the functional and reversible suppression of the hypothalamic–pituitary–gonadal (HPG) axis. During the amenorrheic period, gonadotropins and estradiol levels markedly decreased, which is consistent with hypogonadotropic hypogonadism secondary to energy deficiency. Reduced insulin levels reflected this energy-deficient state ^(10)^, whereas normal thyroid and IGF-1 profiles indicated metabolic adaptation. Chronic low EA reduce leptin, insulin, and IGF-1 signaling, which leads to decreased hypothalamic kisspeptin stimulation and suppression of GnRH pulsatility, as reviewed by Angelidi and cols. ^(11)^. These neuroendocrine adaptations result in reversible inhibition of ovarian function and contribute to altered bone remodeling. Furthermore, these findings illustrate the coordinated neuroendocrine response to chronic low EA, in which diminished GnRH pulsatility leads to temporary suppression of ovarian activity. The restoration of energy balance has been shown to normalize LH pulsatility and menstrual function in exercising women with functional hypothalamic amenorrhea ^(12)^.

This patient had a long history of disordered eating and body image concerns. To further investigate the presence of eating-related disturbances, we applied three validated Portuguese versions of self-administered questionnaires. The Eating Attitudes Test (EAT-26) ^(13)^ which evaluates behaviors that are typically associated with anorexia nervosa and their severity. showed a patient score of 37 points (positive); the Bulimic Investigatory Test of Edinburgh (BITE) ^(14)^, which is aimed at identifying bulimic symptoms and compensatory methods, showed a patient score of 27 points (positive), and the BSQ score ^(15)^, which measures dissatisfaction with body image, showed a patient score of 118 points (negative) Although not diagnostic tools, these questionnaires are widely used to screen individuals at risk for developing eating disorders.

Other screening instruments that are specifically designed for physically active populations, such as the Low EA in Females Questionnaire (LEAF-Q) ^(16)^, have been proposed to identify female athletes at risk for the Female Athlete Triad and related conditions. However, as no validated version of the LEAF-Q was available in Portuguese at the time of assessment, it was not used in this case.

The clinical significance of RED-S extends beyond menstrual function. Impaired bone health poses long-term risks, including increased fracture susceptibility.In this case, evaluation during the amenorrheic period revealed lower than expected areal BMD at the lumbar spine (Z-score below –2 SD), which agrees with reports in the literature on the Female Athlete Triad and functional hypothalamic amenorrhea, where bone loss is frequently observed. Compared with nonathletes, athletes in weight-bearing sports usually have 5%–15% higher BMD. Although weight-bearing exercise is generally associated with improved bone mass, a growing body of research has demonstrated that impaired bone health is common in endurance athletes ^(17)^. For example, as many as 41-45% of elite endurance athletes have low BMD ^(18)^. This apparent paradox is explained by the effects of chronically low EA and menstrual dysfunction, which may override the osteogenic stimulus of physical activity.

Compared with age-matched normal Brazilian women, this athlete also presented lower than expected volumetric BMD and bone structural parameters, as described by Alvarenga and cols. ^(7)^These changes in bone microarchitecture have also been previously documented in the literature. Ackerman and colleagues were pioneers in demonstrating, through HR-pQCT, that athletes with amenorrhea present greater cortical porosity and reduced cortical thickness and density. This occurs, despite preserved or. even increased bone strength, such as higher stiffness and estimated failure load. These findings suggest that exercise may lead to periosteal expansion, but amenorrhea offsets its benefits by favoring endocortical resorption and delays the mineralization of newly formed bone ^(19-21)^

Prolonged hypoestrogenism, as observed during functional hypothalamic amenorrhea, may lead to decreased bone formation and increased resorption and, predispose patients to low bone mineral density and microarchitectural deterioration. The interplay among these hormonal pathways, as summarized in recent reviews ^(11)^, highlights how the restoration of EA and normalization of estradiol are crucial for skeletal health.

Similarly, in a recent study involving long-distance triathletes without evidence of hypoestrogenism, we demonstrated that low EA attenuates the positive skeletal effects of exercise, even in the absence of menstrual dysfunction. Compared with athletes with adequate EA, athletes with low EA had s significantly lower cortical volumetric BMD (tibia), reduced cortical area and thickness, and lower total vBMD (radius). In addition, stress fractures were more frequent in the low EA group (4 out of 12) than in the adequate EA group (2 out of 11), which reinforces the importance of early detection and nutritional management to preserve skeletal integrity in this population ^(22)^

The observed menstrual recovery aligns with findings from the REFUEL study ^(12)^. Furthermore, additional studies highlight that even in athletes with regular menstrual cycles, low EA can compromise bone density and microarchitecture ^(3)^.

A limitation of this study is the absence of leptin measurements, which would have provided an additional biomarker of EA and hypothalamic activation. Moreover, bone mineral density and microarchitectural assessments (DXA and HR-pQCT) were not repeated after menstrual and hormonal recovery.

## CONCLUSION

This case highlights the reversibility of the Female Athlete Triad when EA is restored. The spontaneous remission observed in this triathlete underscores the critical role of exercise modulation and adequate nutritional intake in reversing endocrine disturbances. Preventive strategies, such as regular monitoring of energy availability, menstrual function, and bone health, are essential for mitigating the adverse effects of RED-S and ensuring long-term athletic performance and overall health in female athletes.

## Data Availability

datasets related to this article will be available upon request to the corresponding author.
